# The biological function of *Urtica* spp. and its application in poultry, fish and livestock

**DOI:** 10.3389/fvets.2024.1430362

**Published:** 2024-10-01

**Authors:** Yang Gao, Xuexi Yang, Bo Chen, Huan Leng, Jize Zhang

**Affiliations:** ^1^College of Life Science, Baicheng Normal University, Baicheng, China; ^2^Terra Research and Teaching Centre, Microbial Processes and Interactions (MiPI), Gembloux Agro-Bio Tech, University of Liège, Gembloux, Belgium; ^3^Key Laboratory of Development and Application of Rural Renewable Energy, Biogas Institute of Ministry of Agriculture and Rural Affairs, Chengdu, China; ^4^Institute of Grassland Research, Chinese Academy of Agricultural Sciences, Hohhot, China

**Keywords:** *Urtica* spp., biological functions, action mechanisms, application, animals

## Abstract

*Urtica* species is an angiosperm plant in the Urticaceae family. It serves as a traditional food and medicinal herb, possessing high nutritional value and various bioactive compounds, including polysaccharides, flavonoids, and polyphenolic compounds. In the realm of animal feeds, *Urtica* spp. can replace traditional protein feed sources and high-quality forage, thereby reducing feed costs. Moreover, *Urtica* spp. extract exhibits antioxidant and anti-inflammatory properties and boosts immune regulation. Hence, *Urtica* spp. plays a beneficial role in enhancing animal performance and improving their immune function. Recently, with the development of sustainable farming techniques, the demand for feed additives that prioritize safety, the absence of drug residues, and environmental friendliness have grown. Consequently, *Urtica* spp. and its extracts have received widespread attention in animal production. This article summarizes the biological functions of *Urtica* spp. and its application in animal husbandry while also outlining future prospects for its application. It will provide a scientific basis and reference point for the application of *Urtica* spp. in animal health and breeding.

## 1 Introduction

**Figure 1:**
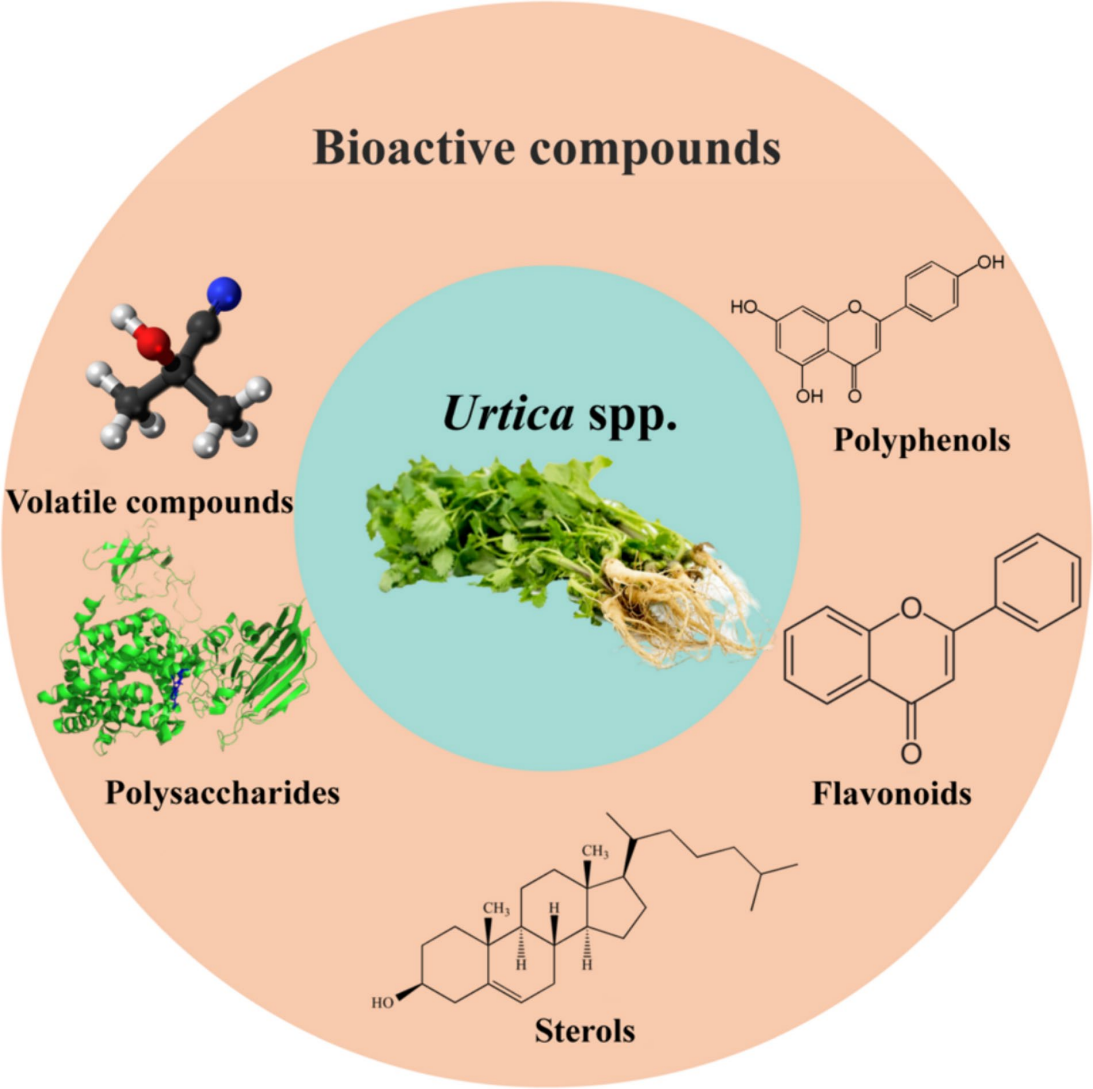
The primary bioactive compounds of *Urtica* spp.

Recently, the heightened emphasis on food safety and nutritional health among human beings has led to a surge in widespread interest in the use of animal feed additives. With the ban on feed antibiotics, the quest for safe and efficient alternatives has gained increasing importance among researchers (1). For agricultural countries, the breeding industry holds a crucial position in the national economy. Owing to the lack of protein resources, essential protein feeds, such as tadpoles and soybean meal, have long relied on imports. Simultaneously, the intensification of environmental problems like global climate change has propelled the protection of the ecological environment and sustainable development into a subject of global concern (2). Under these circumstances, finding a new high-efficiency feed additive to replace traditional protein feeds has emerged as a critical issue for the breeding industry in large agricultural countries. *Urtica* spp., a traditional ingredient and Chinese medicinal material, boasts a rich composition of carotene, protein, cellulose, vitamins, minerals, and an array of biologically active compounds, including polyphenols, flavonoids, polysaccharides, pyrumin compounds, and sterol, as illustrated in Figure 1 (3–5). Research has unveiled that these substances exhibit numerous biological activities such as antioxidant, antibacterial, hypoglycemic, and blood lipid-reducing properties, hence playing an important role in regulating animal immunity and growth performance (6, 7). Furthermore, *Urtica* spp. is characterized by a high protein content and a balanced amino acid profile. The presence of high-quality protein can fulfill the demand for animal growth and development (8). The protein content of *Urtica* spp. is higher compared to soybean meal, while the levels of anti-nutritional factors are low, thus improving the digestive and absorptive abilities of animals (9). Nonetheless, *Urtica* spp. has immense potential for application in animal husbandry. However, the application of *Urtica* spp. as feed components is still in its nascent stage, necessitating in-depth exploration of its application methods and security. Consequently, this article aims to summarize the biological functions, action mechanisms, and application in animal husbandry of *Urtica* spp. with the intention of providing the scientific basis and reference for the utilization of *Urtica* spp. in ensuring the safety of animal production and improving the sustainability of agriculture.

## 2 Overview of the *Urtica* spp

### 2.1 Introduction and geographical distribution of *Urtica* spp

The genus *Urtica* belongs to the family Urticaceae in the major group Angiosperms (flowering plants). The most prominent members of the genus are the stinging nettle *Urtica dioica L.* and the small nettle *U. urens L*., which are native to Europe, Africa, Asia and North America (10). Plants belonging to the genus *Urtica* are herbaceous perennials and can grow up to 2 m tall. Serrated leaves are attached in pairs opposite each other to the stem. The soft leaves and the rest of the plant are coated in hairs, some of which sting. The serrated, hairy leaves and sting are generally recognized characteristics of this plant (11). *Urtica* spp. is a perennial herbaceous plant that serves as an important taxonomic unit in the Urticaceae family. It is highly valued for its versatility in the fields of food, feed, and medicine. Regarding culinary uses, the tender leaves and stems of *Urtica* spp. can be eaten as vegetables that are rich in proteins, vitamins, and minerals. Additionally, nettle tea is a common drink known for its heat-clearing and detoxifying properties. In the field of feed, *Urtica* spp. serves as a valuable source of nutrition for livestock and aquatic animals, promoting growth performance and enhancing immunity. Furthermore, in the pharmaceutical field, *Urtica* spp. is an important Chinese medicinal ingredient with anti-inflammatory properties, often used to treat rheumatism and arthritis (12).

### 2.2 Nutritional and phytochemical composition of *Urtica* spp

Different factors affect the chemical composition of nettle plants, such as the variety, genotype, climate, soil, vegetative stage, harvest time, storage, processing and treatment (13). Stinging nettles are a rich source of nutrients. A comprehensive proximate analysis showed that harvested up-growths contained approximately 90% moisture, up to 3.7% proteins, 0.6% fat, 2.1% ash, 6.4% dietary fiber and 7.1% carbohydrates. On the other hand, nettle leaf powders contain on average 30% proteins, 4% fats, 40% non-nitrogen compounds, 10% fiber and 15% ash (8). The protein content in *Urtica* spp. is well-balanced, with mineral and vitamin levels surpassing other nutrients. Protein can account for 30% of dry matter, with levels in the leaves typically around 23%, in the stems around 14%, and in the roots around 10%. Minerals make up 20% of the dry matter, along with trace elements such as zinc, iron, cobalt, potassium, nickel, and molybdenum and vitamins like vitamin A and vitamin C (14). Research indicates that *Urtica* spp. is abundant in bioactive compounds, including pyrodhumin compounds, flavonoids, carotene, fatty acids, and polyphenols, found in the leaves (15). However, the content of biologically active substances in roots is notably lower compared to leaves, particularly polyphenols. Roots also contain other active compounds like Scopoletin, fatty acids, polysaccharides, and plant hemorchin (16). The seeds mainly included saturated and unsaturated fatty acids, carotene, and *β*-carotene (17). The bioactive compounds present in the leaves, roots, and seeds of *Urtica* spp. are illustrated in Table 1.

**Table 1:** The classification of bio-active compounds in leaves, roots and seeds of *Urtica* spp.

Parts	Bio-active compounds	References
Leaves and roots	Vitamin (A, C, K, B), Minerals (Calcium, Iron, Magnesium, Phosphorus, Potassium, and Sodium), Lipid (Linoleic acid, Linoleic acid, Palm acid, Oleic acid), Amino acid (All essential amino acid), Polyphenols, β-carotene, Grautin.	(3, 17, 68–71)
Seed	Vitamin (A, B, C, E, K), Minerals (Iron, Silicon, Calcium, Magnesium, Manganese, Phosphorus, Potassium), β-carotene, Folic acid, Essential fatty acids	(72–76)

## 3 The biological functions of *Urtica* spp

### 3.1 Antioxidant capacity

Animal oxidation can be triggered by different factors, such as radiation, inflammation, and infection. During the process of oxidative stress, an excess of reactive oxygen species (ROS) and nitric oxide free-radicals (NO_2_) can cause lipid peroxidation, protein oxidation, and DNA damage, thus affecting cellular function (18). Cells eliminate excessive oxygen free radicals through endogenous and exogenous antioxidant defense systems. The endogenous antioxidant defense system comprises superoxide dismutase (SOD), glutathione peroxidase (GSH-P_X_), and cysteine. Exogenous antioxidant defense systems include antioxidant compounds from *in vitro* sources (19, 20). *Urtica* spp. exhibits a range of biological activities, with its antioxidant properties vital for pharmacological activity. Bio-active compounds like flavonoids, polyphenols, phenolic acids, amino acids, and vitamins found in *Urtica* spp. can directly remove free radicals and mitigate oxidative stress reactions (21).

In addition, the bioactive compounds present in *Urtica* spp. can form stable complexes with iron ions through chelation, thereby inhibiting the formation of hydroxyl free radicals to reduce the production of ROS and mitigate cellular oxidative damage (22). Concurrently, the compounds in *Urtica* spp. facilitate the transfer of iron ions, leading to reduced accumulation within the body and decreased oxidative damage to cells (22). Research has demonstrated that leaf extracts of *Urtica dioica* can be chelated through iron ions, resulting in a significant reduction in oxidative stress and lipid peroxidation in the liver and kidney tissues of rats, hence shielding these organs from damage and ameliorating abnormal nerve behavior (23). Additionally, extracts of *Urtica dioica* seed can lower oxidative stress levels in the liver tissues of rats post-radiation exposure, improve their antioxidant capacity, and protect liver tissue from radiation damage (24). Moreover, the antioxidant properties of *Urtica* spp. are closely associated with the NrF2 signaling pathway. Studies have revealed that compounds in *Urtica dioica* can activate the NrF2 signaling pathway, thereby enhancing the activity of antioxidant enzymes (SOD and GSH-P_X_) in the cells and reducing mitochondrial oxidative stress reactions (25). Vajic et al. found that the extracts from *Urtica* spp. leaves can significantly enhance the SOD and GSH-P_X_ activity in the serum of hypertensive rats, leading to lower levels of oxidative stress and protecting cardiovascular health (26). With the addition of *Urtica* spp. extract to the cellular and rat models, the NrF2 signaling pathway is triggered. As a result, the activities of SOD, GSH-P_X_, and glutathione reduction enzymes improve significantly, consequently reducing the level of oxidative stress in macrophages and enhancing the mitochondrial function of myocardial tissues (26, 27). Consequently, *Urtica* spp. exhibits diverse antioxidant properties and can serve as a natural antioxidant in animal husbandry.

### 3.2 Immune regulation

There is a direct relationship between the nutritional supply of animals and their immunity. Improving the nutritional quality of animals has potential value in improving their health (28). Natural nutrients can keep the immune system functioning efficiently. Therefore, improving the immunity of animals can be achieved by improving their nutritional levels (29). *Urtica* spp. has a positive effect on boosting immune function by regulating immune cells. Research has demonstrated that *Urtica* spp. extracts can regulate the function of various immune cells, including macrophages, T lymphocytes, and B lymphocytes (30). The presence of polysaccharides, flavonoids, and alkaloids in *Urtica* spp. can improve the activity of immune cells (31). Moreover, studies have revealed that *Urtica* spp. bolsters its phagocytic ability by stimulating macrophages (32). Furthermore, *Urtica* spp. extracts can promote the proliferation and differentiation of T lymphocytes while simultaneously boosting the production of antibodies by B lymphocytes (33). Additionally, *Urtica* spp. regulates the inflammatory response through MyD88-NF-κB-MAPKs signaling pathways. MyD88 plays an important role in the inflammatory responses of Toll-like receptors (TLR). It can affect the NF-κB pathway and regulate the production of inflammatory factors. MAPKs are an integral component of intracellular signal transformation, including extracellular regulating kinase (ERK), c-Jun NH(2)-terminal kinase (JNK), and P38, which can also participate in regulating inflammatory responses and apoptosis (34). Franciskovic et al. demonstrated that *Urtica* spp. extracts interact with TLRs, utilizing MyD88 as a receptor to regulate the MyD88/NF-κB pathway and inhibiting the phosphorylation of JNK and P38. This inhibition promotes the regulation of the MAPK pathway to reduce the inflammatory response of intestinal epithelial cells induced by LPS (35). Wagner et al. confirmed that polysaccharides derived from *Urtica* spp. can inhibit foot swelling in rats, indicating their anti-inflammatory properties (36). Moreover, *Urtica* spp. extracts can reduce the activation of the NF-κB signaling pathway induced by IL-1β, reducing pain and improving cartilage tissue lesions (37). Consequently, *Urtica* spp. holds promising applications in preventing and treating immune diseases and improving animal immunity, although its mechanisms to improve immune function require further investigation in different animal models.

### 3.3 Anti-bacterial

**Figure 2:**
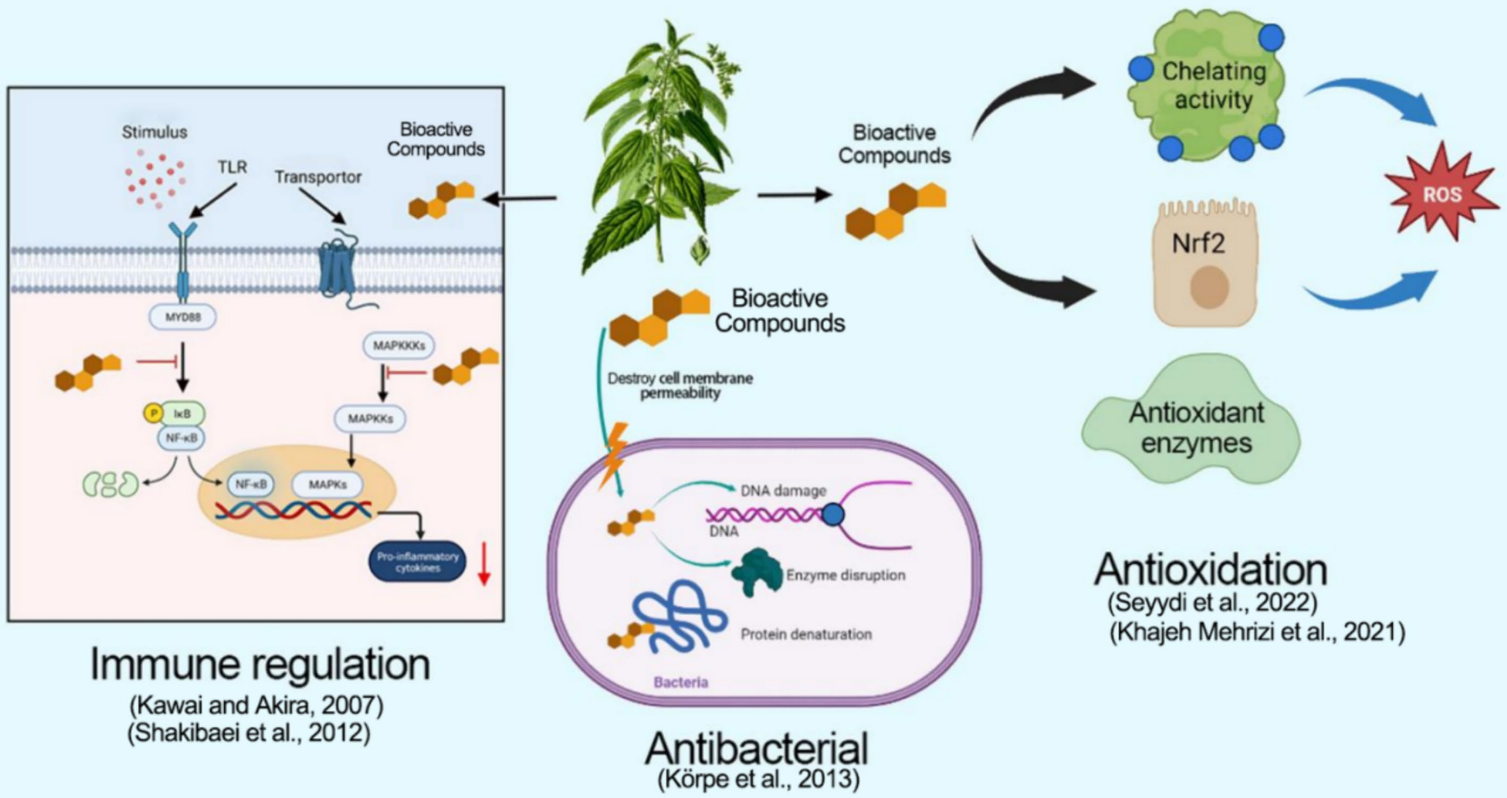
The biological function mechanism of *Urtica* spp.

With the ban on antibiotics, more and more natural active substances have been discovered to replace antibiotics (38). Improving the antibacterial ability of animals can improve their growth performance (39). *Urtica* spp. extracts exhibit a broad spectrum of antibacterial, anti-fungal, and anti-viral properties. The inhibition of microbial growth is attributed to the synergistic action of multiple bioactive constituents. Research has demonstrated that the addition of 150 g/mL *Urtica* spp. extracts can effectively suppress the growth of *Bacillus*, *Golden Polychiococcus*, *Salmonella,* and *Viblus*. Notably, the key bioactive compounds that are responsible for this activity are flavonoids (40). *Urtica* spp. also contains various phenolic compounds, like cinnamonic acids, which display a significant inhibitory effect on *Pseudomonas fragi* and *Campylobacter jejuni* (41). Furthermore, the widespread polysaccharides in *Urtica* spp. exhibit inhibitory effects on foodborne and plant-pathogenic bacteria (42). Studies have revealed that the positively charged components in *Urtica* spp. can interact with negatively charged components on the microbial cell membrane, leading to changes in its permeability, causing hydrolysis, and subsequent binding with DNA in microbial cells, thus inhibiting microbial protein synthesis. This inhibition results in protein degeneration or loss of enzyme activity, ultimately causing bacteriostatic effects (43). *Urtica* spp. extracts can also inhibit bacterial biofilm formation, consequently reducing bacterial resistance. Research indicates that adding 2–20 mg/mL *Urtica* spp. extract can inhibit the biofilm formation in *Salmonella*, thereby enhancing bacterial sensitivity to antibiotics (44). Recently, nanoparticles derived from *Urtica* spp. can disrupt cell membranes, causing microbial membrane damage and inhibiting the growth of bacteria and fungus cells (45, 46). Furthermore, the latest research has identified the separated urticine cohesion in the root system of *Urtica* spp. as a monomeric protein that can bind to the spike protein on the surface of the COVID-19 virus, thus inhibiting the CoV-2 variants (47). In conclusion, the primary biological function of *Urtica* spp. is anti-oxidation, immune regulation, and antimicrobial activity. The mechanism of its function is illustrated in Figure 2.

## 4 The application of *Urtica* spp. in animal husbandry

**Figure 3:**
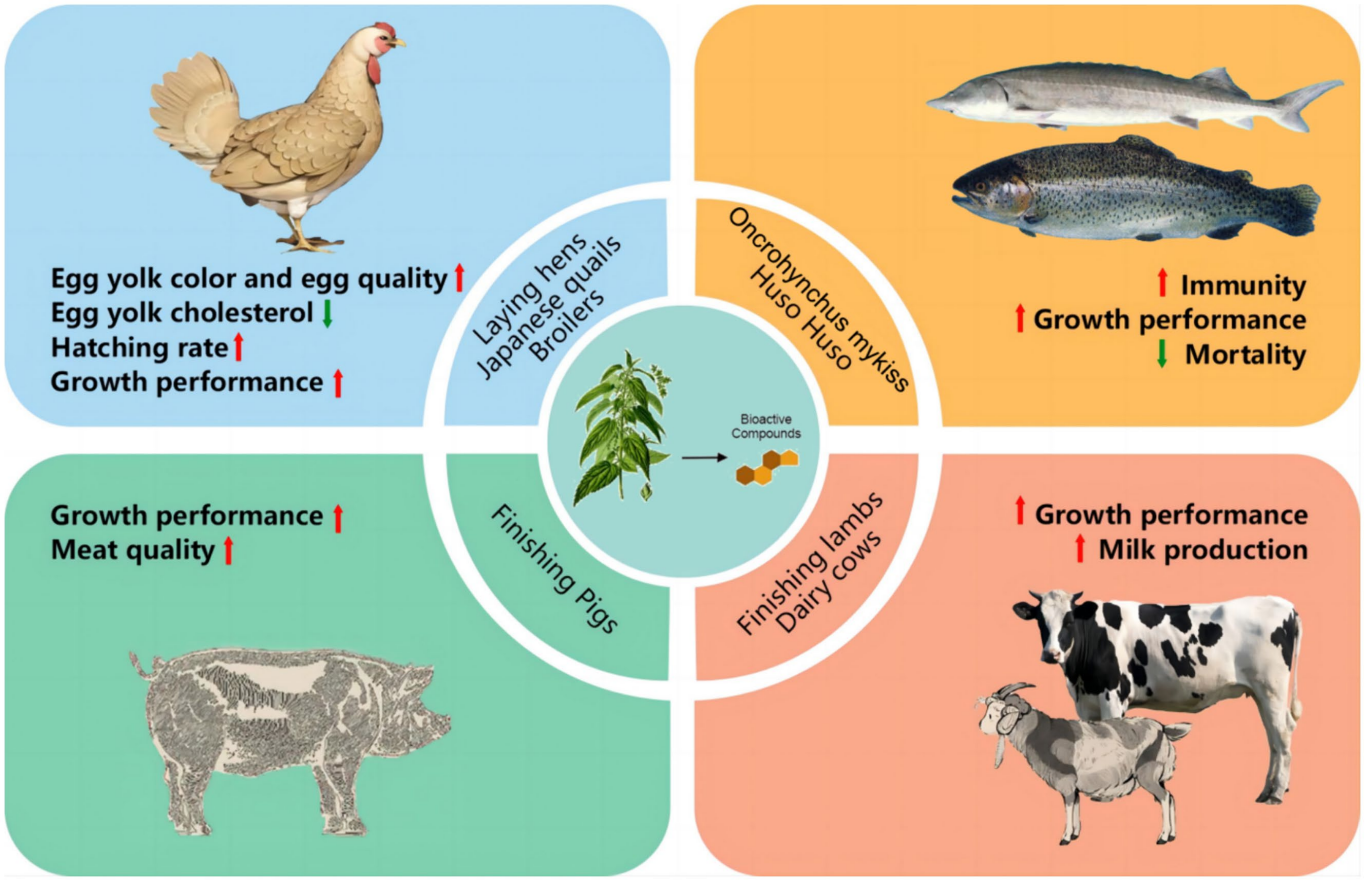
The application of *Urtica* spp. in animals.

Currently, *Urtica* spp. is mainly used in feed for poultry, aquatic animals, ruminants and pigs. The dosage and effects of *Urtica* spp. in different species are detailed in Table 2 and Figure 3.

**Table 2:** The dosage and effects of *Urtica* spp. application in animals.

Species	Dose (g/kg)	Effects
Laying hens	6.25–25	Improve egg yolk color and egg quality
Japanese quails	1–6	Reduce egg yolk cholesterol and improve hatching rate
Broilers	0.5–1.5	Improve growth performance
Rainbow trout	0.1–0.5	Reduce mortality and improve growth performance
*Huso Huso*	3–12	Improve immunity
Finishing lambs	250–500	Improve growth performance
Dairy cows	50–100	Increase milk production
Finishing pigs	500–1,000	Improve growth performance and meat quality

### 4.1 Application in poultry production

Loetscher et al. discovered that supplementing 6.25, 12.5, and 25 *Urtica* spp. to 70-week-old laying hens, respectively, resulted in enhanced egg yolk color with the increasing concentration of *Urtica* spp. after 4 weeks of trial time. Similar effects were observed in the group that added artificial pigmentation; however, the content of natural vitamin E in the egg yolk in the *Urtica* spp. group was significantly higher than the CON group without any negative effects on the productivity and quality of eggs of laying hens (48). Similarly, 15% *Urtica* spp. was supplemented with 28-week-old laying hens as a substitute for soybean meal. The results demonstrated a notable increase in the n-3 polyunsaturated fatty acids, egg yolk color, and eggshell thickness, along with reduced cholesterol levels in the serum and yolk (49). Moula et al. (50) also revealed that adding 6% of *Urtica* spp. to the diet of 10-week-old Japanese quail led to a significant decrease in the level of yolk cholesterol, blood cholesterol, and triglyceride levels without any adverse effect on productive performance. Furthermore, in older Japanese quails (52 weeks old), adding *Urtica* spp. to the basal diet not only increased egg production but also increased reproductive and incubation rates (51). Evidently, the addition of *Urtica* spp. to poultry diets has a positive impact on improving egg quality, lipid metabolism, and egg production performance.

Ahmadipour et al. (52) added 0.5, 1, and 1.5% *Urtica* spp. to the diet of 1-day-old broilers (Ross308) in high-altitude regions. The results revealed a significant increase in relative overexpression of catalase (CAT) and superoxide dismutase 1 (SOD1) in the liver and lung of the chickens that were fed *Urtica* spp. Lipid peroxidation was significantly suppressed, as reflected in reduced levels of malondialdehyde (MDA) in the circulatory system. Adding 4% *Urtica* spp. to the broiler diet mitigated cortisol levels, total cholesterol concentration, and tissue damage markers such as creatine kinase induced by heat stress (53). In standard breeding conditions, adding 0.5 g/kg root extract of *Urtica* spp. or 0.05% nanoencapsulated *Urtica* spp. resulted in enhanced growth performance parameters such as body weight gain, feed conversion rate, and carcass weight (54). In conclusion, supplementing poultry diets with *Urtica* spp. can substitute soybean meal, improve egg quality, boost stress resistance, and promote growth performance.

### 4.2 Application in aquatic animals

Microbial infection is the primary cause of the slow growth and death of aquatic animals. *Urtica* spp. serves as a growth promoter and immune booster, widely used in the intensive aquaculture industry. Rainbow trout currently stands as the main aquaculture fish globally, boosting an annual production of up to 762,000 tons with high economic value. It has emerged as the main research focus of *Urtica* spp. in aquaculture applications. Studies have indicated that feeding rainbow trout with 1% *Urtica* spp. for 14 days led to decreased mortality following exposure to *Aeromonas hydrophila*. Furthermore, there was a significant enhancement in serum bactericidal activity, respiratory burst, and lysozyme activity in the treatment groups compared to the control group (32). Saeidi et al. demonstrated that adding 3% *Urtica* spp. to the rainbow trout diet significantly improved hematological parameters while improving body growth and feed conversion rate. The mortality rate is significantly lower compared to the control group (55). Bilen et al. manifested that adding 0.1 and 0.5 g/kg *Urtica* spp. to the rainbow Trout diet, respectively, resulted in higher fish weight and specific growth rate. The 30-day feeding trial revealed that all treated groups outperformed the control group when challenged with the bacterial pathogen *A. hydrophila* (56). These findings suggest that adding *Urtica* spp. to the diet can improve the growth performance and immune function of rainbow trout. Beluga (*Huso Huso*) is a large sturgeon fish with high economic value. Due to excessive fishing in recent years, its wild resources are now endangered. Presently, the population is primarily maintained through artificial reproduction, yet microbial infections in the breeding process can lead to substantial losses. Binaii et al. investigated the effects of adding 3, 6, and 12% *Urtica* spp. to the diets of juvenile Beluga, respectively. After an eight-week trial period, the level of neutral granulocytes and hemoglobin in the blood increased significantly compared with the control group, with these indicators directly related to the levels of *Urtica* spp. supplementation. The group fed with 12% exhibited a highly significant difference in total red blood cells, total protein, and total immunoglobulin (57). Research has demonstrated that adding *Urtica* spp. to the basal diet can enhance thermal, biochemical, and immune function in fish. This can be attributed to the following reasons: First, *Urtica* spp. is rich in vitamin C and iron. Vitamin C facilitates iron absorption in the fish intestine, thereby improving blood biochemical indicators. Second, the flavonoids present in *Urtica* spp. can activate neutral granulocytes and produce ROS to play a bactericidal effect. Third, the feed of *Urtica* spp. can increase immunoglobulin by increasing the total protein levels of fish (58). In conclusion, adding an appropriate amount of *Urtica* spp. to the diet can significantly enhance the growth performance and immune function of various fish species; however, the mechanism still needs to be further explored.

### 4.3 Application in the production of ruminants

The addition of *Urtica* spp. to the sheep diet can promote growth performance and improve nutritional metabolism. By adding 250 and 500 g/kg *Urtica* spp. to the diet of finishing lambs, respectively, an increase in blood glucose level was observed, accompanied by a decrease in total cholesterol and triglyceride levels. The antioxidant parameters had also improved significantly. The contents of unsaturated fatty acids in the treatment group were significantly higher than the control group, particularly in the 500 g/kg group (59). The addition of 250 and 500 g/kg *Urtica* spp. to diets of growth lamb led to a significant increase in the apparent digestion of crude protein, neutral, and acidic washing fibers compared to the control group. This increase could be attributed to the presence of saponin in *Urtica* spp., which can stimulate the smooth muscle contraction of the abomasum and secrete digestive enzymes that improve the efficiency of the digestion and absorption of feeds (60). Feeding sheep with urticaries changes the composition of the microbiota, decreases the pH rumen value, and elevates propionic acid and volatile fatty acid concentrations, thus enhancing the production performance of sheep (59, 61). Furthermore, adding 12% *Urtica* spp. to the dairy cow diet as a substitute for *lucern* has been shown to have no detrimental effect on production performance. It improves the composition of fatty acids and amino acids, immune function, and antioxidant capacity (62). Humphries et al. added 50 and 100 g/kg *Urtica* spp. to the diet of dairy cows instead of ryegrass. The findings indicated no adverse effect on milk production while also regulating the pH value of the rumen to effectively prevent ruminal acidosis that may be induced by high-grain diets (63). In conclusion, *Urtica* spp. can be used as a substitute for pastoral grass. It can not only reduce breeding costs but also enhance the production performance of ruminants. The bio-active substances can also play a significant role in enhancing immunity and antioxidant capabilities.

### 4.4 Application in the swine industry

Szewczyk et al. added 500 and 1,000 mg/kg of *Urtica dioica* extract in the dietary phases of fattening pigs (60–112 kg), respectively. The results demonstrated an increase in the loin eye area, with the supplementation with *Urtica dioica* affecting the fatty acid profile in the loin fat. This led to a reduction in short-chain fatty acids and an elevation in the proportion of multi-unsaturated fatty acids (64). By adding *Urtica* spp. to the basal diet of finishing pigs (60–110 kg), the protein content in the longissimus muscle increased while the fat content decreased in the treatment group compared to the control group. Additionally, the supplementation of *Urtica* spp. also increased the lightness of meat and maintained the color stability for 6 months when stored at 20°C. Furthermore, it slightly improved the meat’s oxidative stability during frozen storage and boosted polyunsaturated fatty acid (PUFA) contents, mainly due to reducing monounsaturated fatty acid (MUFA) contents (65). The addition of 500 mg/kg *Urtica* extract to the diets of fattening pigs (60–112 kg) resulted in a significant improvement in the meat quality and a reduction in cholesterol content in the pork; however, it displayed no significant impact on the growth performance (66). Supplementing *Urtica* extracts to the diet of piglets can significantly improve growth performance, with significantly higher villi height of the ileum in the treatment group compared to the control group (67). In conclusion, *Urtica* spp. can serve as feed additives to improve growth performance during the growing stage and meat quality during the fattening stage of pigs. However, there is limited research on using *Urtica* spp. in the swine industry, especially concerning local breeds. Consequently, the mechanism of *Urtica* spp. needs to be explored further in terms of its role in regulating pork meat quality.

## 5 Conclusion and perspectives

*Urtica* spp. is being considered a substitute for traditional protein feed and high-quality pasture due to its abundance of various nutrients and bio-active compounds, thereby reducing the cost of feed. Furthermore, it also possesses antioxidant, anti-inflammatory, and immune-regulating properties, which positively impact animal production. Presently, research on *Urtica* spp. primarily focuses on food processing and pharmacological studies. However, there are still some limitations in the use of *Urtica* spp. For example, excessive consumption of *Urtica* spp. can cause damage to the respiratory system and skin of animals. Therefore, the appropriate additive dosage in animal feed is very important. At the same time, the literature in the field of animal nutrition is limited, necessitating a thorough exploration of the key bio-active constituents in *Urtica* spp. In the future, the application of *Urtica* spp. should be expanded to replace traditional protein feed and high-quality pastoral grass in animal production, particularly regarding safety alternatives and growth performance. Regarding the extraction of *Urtica* spp., the key bio-active compounds and their mechanisms in animals should be further explored to establish the theoretical basis for the application of *Urtica* spp. in animal husbandry.
